# Visualization of four-dimensional X-ray absorption fine structure data using a virtual reality system

**DOI:** 10.1107/S1600577524011226

**Published:** 2025-01-01

**Authors:** Haruo Igarashi, Daiki Kido, Yutaka Ishii, Yasuhiro Niwa, Atsushi Okamoto, Masao Kimura

**Affiliations:** a School of Advanced Science and Engineering Waseda University Okubo Shinjuku-ku Tokyo169-8555 Japan; b Institute of Materials Structure Science High Energy Accelerator Research Organization 1-1 Oho Tsukuba Ibaraki3050801 Japan; c The Graduate University for Advanced Studies (SOKENDAI) 1-1 Oho Tsukuba Ibaraki3050-801 Japan; d Department of Mathematics Kyushu University Motooka Fukuoka819-0395 Japan; e Graduate School of Environmental Studies Tohoku University Sendai Miyagi980-8579 Japan; Advanced Photon Source, USA

**Keywords:** spectromicroscopy, multi­dimensional data, virtual reality, X-ray absorption fine structures, *4D-XASView*

## Abstract

X-ray spectromicroscopy is extensively utilized for nondestructive mapping of chemical states in materials. However, understanding and analyzing the geometric and topological aspects of such data pose challenges due to their representation in 4D space, encompassing (*x*, *y*, *z*) coordinates along with the energy (*E*) axis and often extending to 5D space with the inclusion of time (*t*) or reaction degree. In this study, we addressed this challenge by developing a new approach and introducing a device named ‘*4D-XASView*’, specifically designed for visualizing X-ray absorption fine structures (XAFS) data in 4D (comprising 3D space and energy), through a multi-projection system, within the virtual reality (VR) environment. As a test case for the new system, X-ray spectromicroscopy measurements were conducted on a specimen prepared from serpentinized harzburgite sourced from the upper mantle section of the Oman ophiolite. Our *4D-XASView* facilitates the visualization and analysis of the geometric and topological aspects of the data using VR goggles, enabling detailed exploration of microstructures via rotation and zooming functionalities. This capability allows us to extract XAFS spectral data by selecting specific positions and regions, thereby aiding in the identification of ‘trigger sites’ (magnetite in serpentine), which are characteristic locations within materials that substantially influence the macroscopic propagation of reactions. Our methodology establishes a new platform for analyzing 4D or 5D XAFS data that has applicability potential in various other multidimensional datasets, including microstructures coupled with spectroscopy and diffraction data.

## Abstract 2

4D imaging X-ray absorption fine structure data were successfully visualized in virtual reality space. The proposed method provides a novel approach for analyzing the geometric and topological nature of multidimensional spectroscopic data.

## 1. Introduction

Spectromicroscopy, which involves the acquisition of 2D or 3D images of materials at varying X-ray energies, has gained prominence owing to recent advancements in X-ray optics and measurement techniques. It is extensively utilized for nondestructive mapping of chemical states in materials (Jacobsen, 2020 ▸; Wachs & Bañares, 2023 ▸). In particular, the X-ray absorption fine structure (XAFS) is frequently employed as a spectroscopic technique owing to its ability to provide diverse information. This includes details about the chemical state of absorbing atoms (such as valence) as well as the coordination states of neighboring atoms or ligands (Bunker, 2010 ▸; Van Bokhoven & Lamberti, 2016 ▸). Moreover, XAFS measurements can be performed nondestructively, preserving the specimen from exposure to air. This method has found extensive applications for observations *in situ*, allowing the observation of changes in chemical states within materials under various working conditions such as high temperatures and gaseous environments, or under different electrochemical potentials.

For example, 2D chemical-state mapping, known as imaging XAFS, has been successfully utilized in studies on the redox reaction in lithium-ion batteries [FeLiPO_4_ (Katayama *et al.*, 2014 ▸)] and reduction of iron-ore sinter [Fe–Ca–O (Kimura *et al.*, 2017 ▸, 2018 ▸)], among others. Mapping the chemical states within specimens in 3D with a high-spatial-resolution <50 nm, known as XAFS computed tomography (XAFS-CT) or transmission X-ray microscopy (TXM), has been employed for nondestructive and *in situ* observations of materials under various working conditions. This includes the study ofchanges in metallic chemical states within lithium-ion batteries during charge–discharge cycles (Meirer *et al.*, 2011 ▸; Xu *et al.*, 2017 ▸), degradation of environmental barrier coating (Yb–Si–O) (Takeichi *et al.*, 2018 ▸), reduction of iron-ore sinter [Fe–Ca–O (Kimura *et al.*, 2017 ▸)] and others.

**Figure 1:**
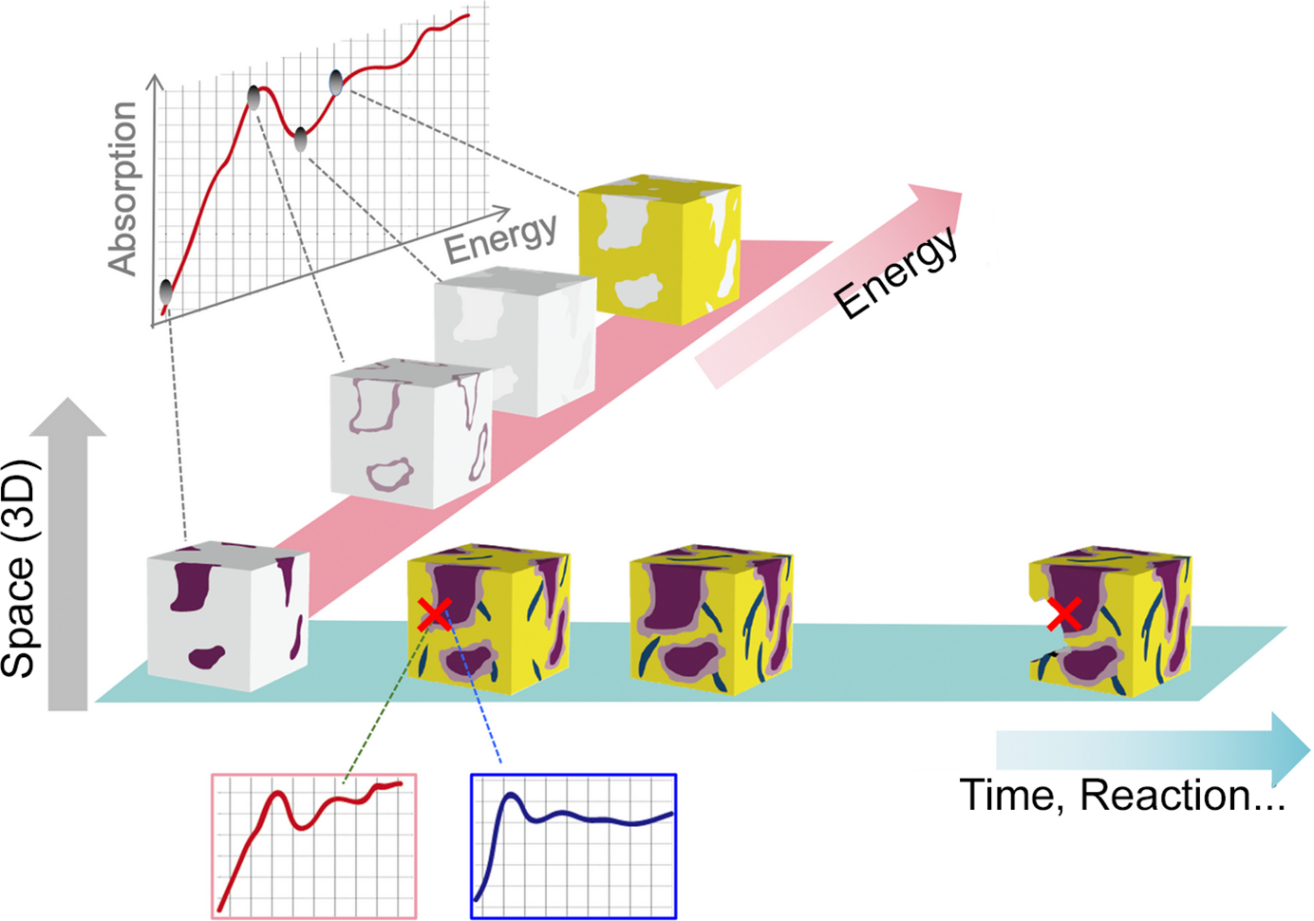
Schematic of 5D XAFS data (3D space + energy + time) obtained through the use of analytical methods in spectromicroscopy, such as XAFS-CT or TXM.

In these experiments, 3D or 4D XAFS data (3D space + energy) were obtained, often reaching sizes on the order of several tens of gigabytes (GB) or terabytes (TB) (Fig. 1 ▸). Consequently, the analytical approach for 4D and big data poses prominent challenges for researchers aiming to intuitively visualize and understand these data. Thus far, only part of the 3D or 4D data has been extracted based on human visualization (*i.e.* reduction of dimensions), and 2D or 3D viewing approaches or programs are used for intuitively comprehending the data. Another analytical approach is the reduction of the energy axis in 3D or 4D data by linear-combination-fitting, assuming that the chemical state of each voxel can be expressed by the combination of those of standard specimens. However, this analytical approach could miss the possibility of finding unknown chemical states, and we need new analytical tools for simultaneous 3D viewing and confirmation of XAFS spectra. Thus, extracting the complete information included in big and higher-dimensional data remains challenging, indicating a new approach is required for identifying ‘trigger sites’ that represent specific locations within materials and dictate macroscopic properties or reactions (Kimura *et al.*, 2018 ▸).

Moreover, X-ray absorption near-edge structure (XANES) data derived from XAFS-CT volume measurements are often lower quality compared with bulk-averaged or 2D imaging XAFS data owing to limited measurement times and 3D reconstruction from 2D absorption images. Consequently, the segmentation process, which involves fitting XANES data with reference materials, can yield inaccurate results. Thus, segmentation verification via simultaneous examination of the XANES spectrum and 3D microstructure is essential. The need for such a system led to the development of the *4D-XASView* program.

The concept of higher-dimensional data is essential in materials science as well as various fields of natural sciences and mathematics, and, to date, a plethora of mathematical approaches has been developed and assessed to understand higher-dimensional data. However, it remains challenging for humans to intuitively comprehend these higher-dimensional objects, even in 4D (Ambinder *et al.*, 2009 ▸; Wang, 2014 ▸). Recent developments in computer graphics and virtual reality (VR) technologies have been used to visualize 4D figures or interact with them (Aguilera, 2006 ▸; Miwa *et al.*, 2018 ▸; Matsumoto *et al.*, 2019 ▸; Igarashi & Sawada, 2023 ▸). One of the most comprehensive approaches is to project a 4D figure into the 3D virtual space using 4D rigid-body rotation and then observe it from multiple viewpoints. Note that, unlike 3D space, wherein rotation has only three degrees of freedom, rotation in 4D space involves six degrees of freedom. Therefore, assigning each degree of freedom to individual operations by VR controllers is a highly nontrivial problem (see Section 3).

However, these VR technologies for 4D data visualization cannot be simply applied to visualize 3D or 4D (3D space + energy) XAFS data – the focus of our study – because of the uniqueness of these 3D/4D XAFS data compared with simple 4D data, wherein each axis is equally independent. The meaning of energy dimensions is different from that of 3D space. Taking into account these unique features of 4D XAFS data, we have developed a novel approach and implemented a device named *4D-XASView*. This device can visualize the 4D (space + energy) and large-scale XAFS data using VR space. Our approach and device have been developed based on the multi-projection system in a VR system called *Polyvision* (Matsumoto *et al.*, 2019 ▸).

## 2. Materials and methods

### 2.1. Development of *4D-XASView* system

**Figure 2:**
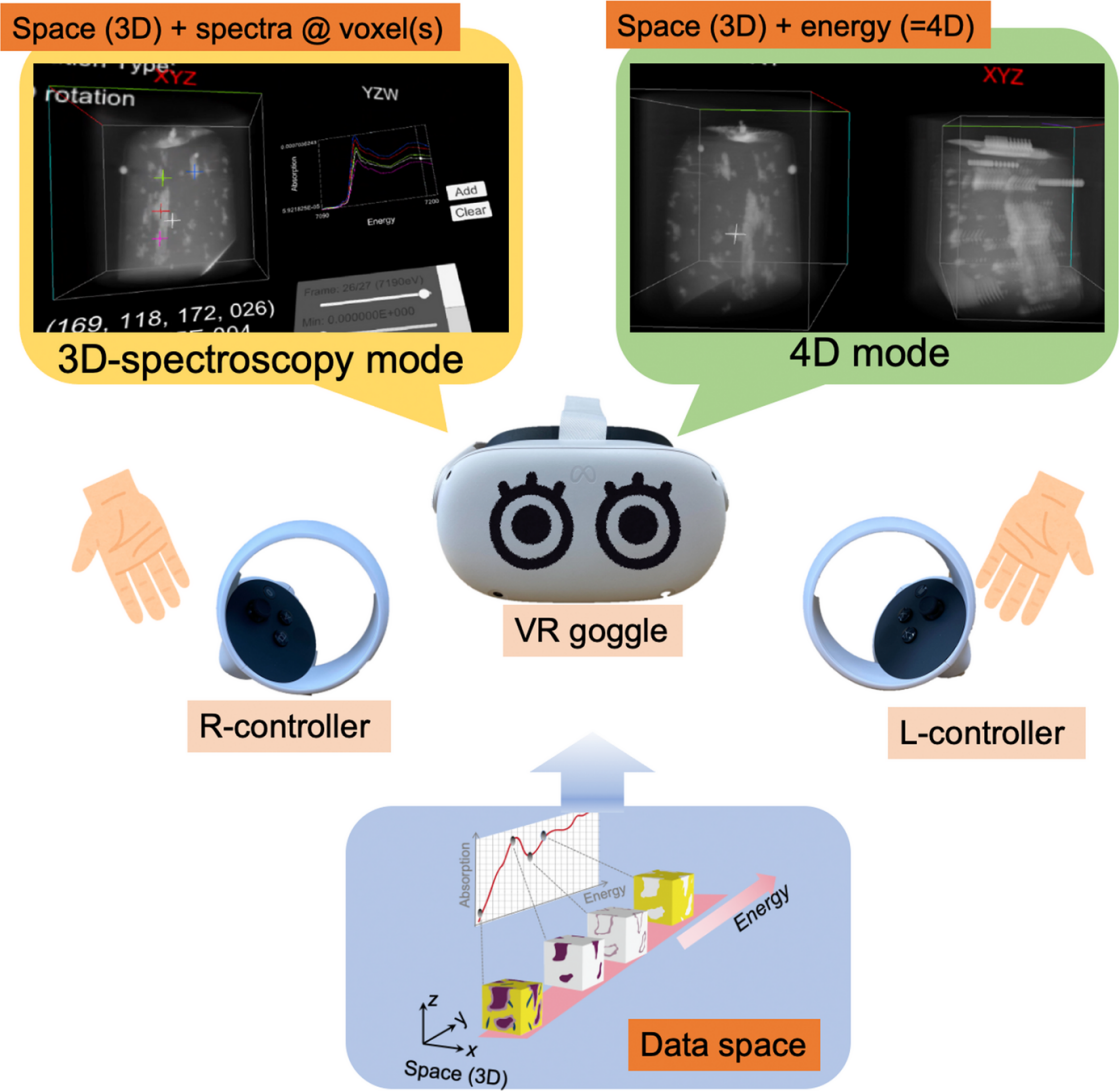
Overview of the *4D-XASView* system developed.

The new *4D-XASView* program was developed to visualize and analyze 4D XAFS data using an exploration system designed for intuitive understanding of 4D space (Igarashi & Sawada, 2023 ▸) with the assistance of *Polyvision* (Matsumoto *et al.*, 2019 ▸). Fig. 2 ▸ provides an overview of the developed system. *4D-XASView* facilitates exploration of 4D XAFS data via three modes: (*a*) 3D volume data view, (*b*) 3D spectroscopy (3D-spec.) and (*c*) 4D. The system operated within the *Unity* software environment (Unity Technologies, Inc.) and the data were visualized using VR goggles; specifically Meta Quest 2 (Meta Platforms, Inc.) goggles were used and connected via a USB-C cable to a laptop computer.

This system has been designed to integrate the capability of displaying volumetric 4D XAFS data into *Polyvision*, which previously dealt primarily with point clouds. The data were treated as stacks of 3D textures containing X-ray energy coordinates, and each texture was rendered using volume ray-casting (Balsa Rodríguez *et al.*, 2014 ▸) after it underwent 4D geometric transformations. Moreover, a series of menus has been incorporated for adjusting display methods and presenting spectra, along with controls that enhance the intuitiveness of rotational operations (Igarashi & Sawada, 2023 ▸).

### 2.2. Specimen

As a test case for the new system, *4D-XASView*, X-ray spectromicroscopy measurements were conducted on a specimen prepared from serpentinized harzburgite sourced from the upper mantle section of the Oman ophiolite, obtained from drill site CM1A of the Oman Drilling Project (Yoshida *et al.*, 2023 ▸). When seawater infiltrates the oceanic lithosphere, mantle rocks like harzburgite [(Mg,Fe)_2_SiO_4_ + (Mg,Fe)_2_(Si,Al)_2_O_6_] react with seawater, leading to the formation of serpentine [(Mg,Fe)_3_Si_2_O_5_(OH)_4_], brucite [Mg(OH)_2_] and magnetite (Fe_3_O_4_). This process, known as serpentinization, has gained considerable attention owing to the production of H_2_ through iron oxidation (Klein *et al.*, 2013 ▸). Consequently, the chemical state of iron [Fe(II) or Fe(III)] present in rocks and minerals serves as a valuable indicator of redox reactions, making the mapping of its chemical state essential for understanding the reaction mechanism.

The specimen was sourced from an upper-mantle region composed of harzburgite, where olivine grains displayed serpentinization with mesh-like fractures filled with serpentine, brucite and magnetite. A pillar measuring approximately 20 µm in diameter and 20 µm in length was extracted from the rock samples using a focused ion beam after identifying regions of interest based on electron probe microanalysis.

### 2.3. Spectromicroscopic measurements

Spectromicroscopic measurements were performed using the XAFS-CT technique with an X-ray microscope [Xradia Ultra, Carl-Zeiss X-ray Microscopy, Inc. (Kimura *et al.*, 2018 ▸; Niwa *et al.*, 2019 ▸)] installed in the NW2A beamline of Photon Factory Advanced Ring (PF-AR) in the Institute of Materials Structure Science (IMSS) at the High Energy Accelerator Research Organization (KEK) in Japan. A monochromatic X-ray beam was focused onto the sample via an elliptical glass capillary. The transparent image was then projected onto the scintillator using a Fresnel zone plate lens, providing a magnification of approximately 70× at an X-ray energy of 8 keV. Subsequently, the image was further magnified by an optical lens (∼20×) and captured using a charge-coupled device camera. The overall spatial resolution achieved was ∼50 nm. Detailed information about the equipment setup can be found elsewhere (Kimura *et al.*, 2018 ▸; Niwa *et al.*, 2019 ▸).

X-ray computed tomography (X-CT) measurements were conducted by changing the X-ray energies. Projection images were acquired using a zone plate with a diameter of 100 µm and an outermost zone width of 30 nm. The voxel size for reconstruction was set at 24.4 nm, but it was binned to 48.8 nm for analysis purposes. Each tomography involved 361 projections (0.50° per slice) and was repeated at 28 energy points in the range 7090–7200 eV (around the Fe *K*-edge). This included a 5 eV step in the pre-edge region, 2 eV around the absorption edge and 10 eV in the post-edge region. Although the energy steps were large, our initial analysis confirmed that these conditions were sufficient to distinguish the chemical states of iron in this case. We performed X-ray microscopy measurements according to the minimum requirements to obtain the test data for the development of the program. The exposure time for each slice was 2 s, and the total acquisition time for gathering volume data at different energies was approximately 12 h. The acquired data underwent analysis using the TXM *Wizard* software (Liu *et al.*, 2012 ▸). The *x*-, *y*- and *z*-axis positions of reconstructed 3D images at different X-ray energies were aligned based on image-processing techniques. Subsequently, 4D XAFS data were obtained as stacks of X-ray absorption image data in 3D at varying X-ray energies, including that of Fe *K*-edge, with the data size reaching 25 GB. The absorption values for different X-ray energies at a specific (*x*, *y*, *z*) coordinate corresponded to XAFS spectra at those positions.

For the development of the new system, test data were derived from raw data by compressing cross-sectional images from 1024 × 1024 to 256 × 256 using the software program *ImageJ* [version 1.53k, National Institutes of Health, Bethesda, MD, USA (Schneider *et al.*, 2012 ▸)]. This reduction of the data size by binning ensured real-time and smooth image movement within VR space, even when using a PC with standard processing power. The program can read ‘energy stack’ files from XAFS-CT measurements, consisting of X-CT data stacks at different X-ray energies, with each X-CT datapoint containing cross-sectional images in TIFF format. Reference XANES spectra at the Fe *K*-edge were also measured using iron oxides and metal with a transparent geometry at the BL-9C beamline of the PF in IMSS, KEK, Japan.

## 3. Results: features of the *4D-XASView* program developed for 4D XAFS data visualization

We can explore the 4D XAFS data using *4D-XASView* via three modes. The proposed system provides direct visualization of 4D XAFS data within a VR environment. Initially, the XAFS data were presented as overlays of volumetric X-CT data corresponding to each X-ray energy. This setup allows users to manipulate and observe the data from preferred angles, simulating a physical handling experience, and facilitates analysis of specific spatial regions.

Furthermore, by applying a 4D rigid-body rotation to the XAFS data, a geometric structure incorporating the three spatial dimensions and the energy dimension was generated. Users can explore this 4D structure through multiview orthographic projections and intuitive rotational controls, facilitating the identification of ‘trigger sites’ that may be overlooked when a conventional 3D viewer is used. The distinctive features of *4D-XAFSView* are outlined in this chapter. Additionally, refer to Movies S1 and S2 of the supporting information for a recording showcasing the analysis of 4D XAFS data using VR goggles.

### 3.1. 3D volume data view mode

**Figure 3:**
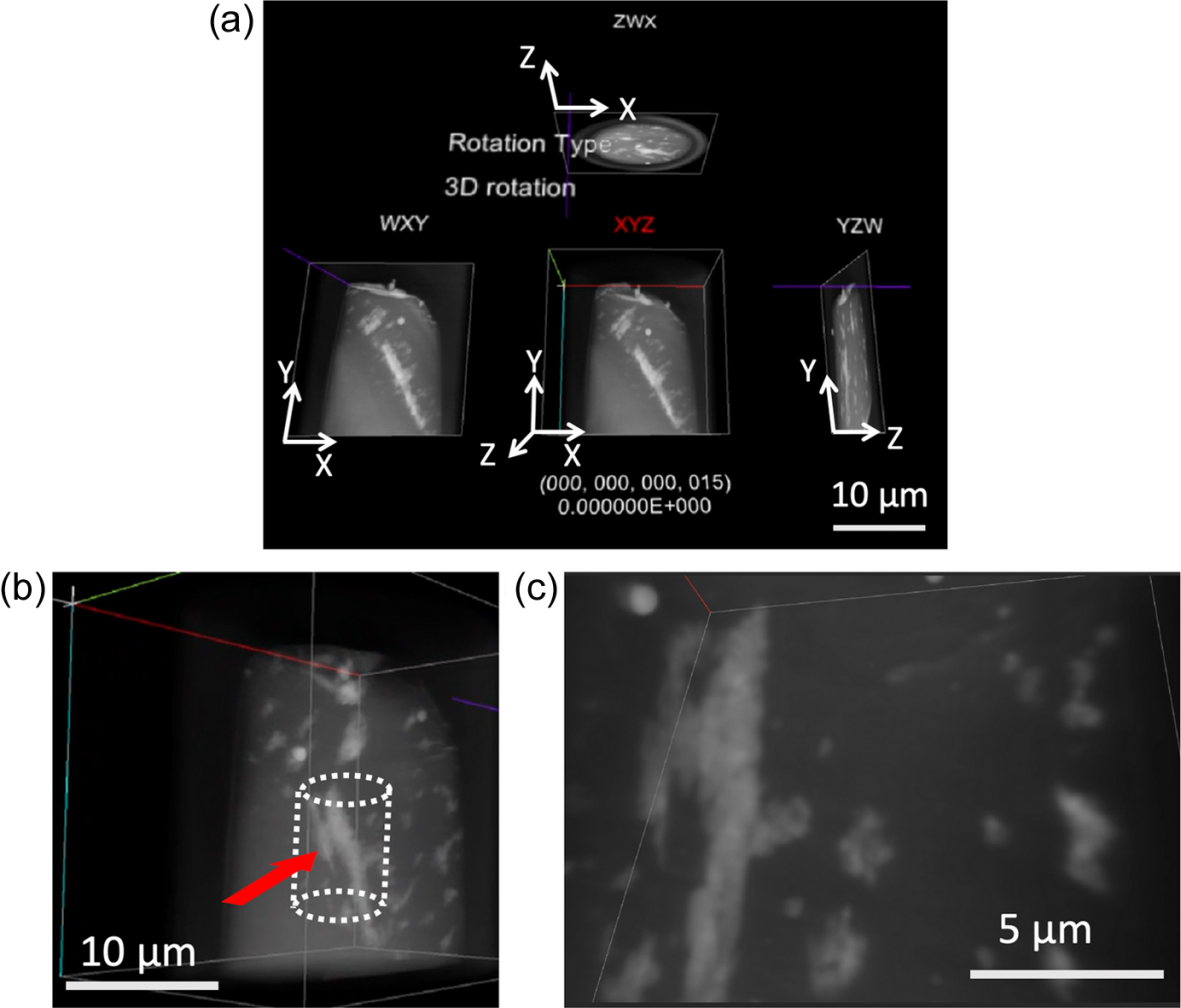
Views of the 3D volume data view mode in VR space using *4D-XAFSView*. (*a*) Illustration of the specimen in VR space. The central view (indicated by *XYZ*) represents the 3D transparent absorption image at a specific X-ray energy. The left, right and top views depict the *Z*-, *X*- and *Y*-axis projections, respectively. (*b*) Magnified image of a part from the central image in (*a*). (*c*) Perspective of the observer within the VR environment. The observer can navigate inside the specimen from the arrow direction in (*b*) to explore and observe. Refer to Movie S1 of the supporting information for further details.

In this mode, we can observe the microstructural details of the 3D volume data typically obtained through X-CT. Fig. 3 ▸ illustrates typical specimen views within VR space at an X-ray energy of 7128 eV, approximately 27 eV above the Fe *K*-edge. The central view (indicated by *XYZ*) represents the 3D transparent absorption image at a specific X-ray energy. Meanwhile, the left, right and top views display the *Z*, *X* and *Y*-axis projections, respectively.

**Figure 4:**
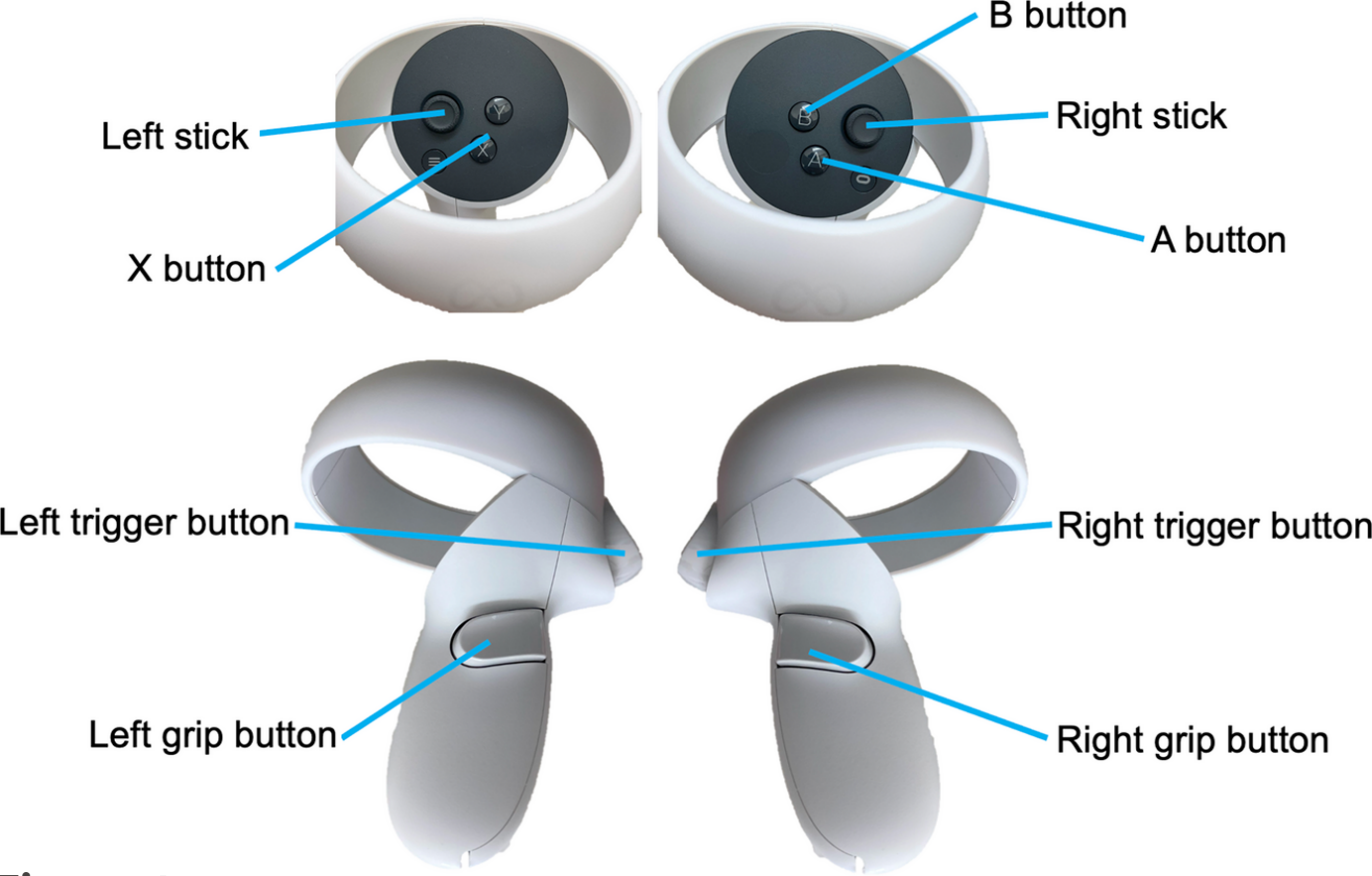
Motion controller of Meta Quest 2 and its buttons for image control in VR space.

To navigate the images within VR space, two motion controllers (refer to Fig. 4 ▸) were utilized as outlined below (also refer to Movie S1 of the supporting information):

(i) Selection of the X-ray energy: the control stick at the right-hand controller or the frame in the panel (mentioned later).

(ii) Rotation of the specimen: rotation of the left-hand controller while pressing the left grip button.

(iii) The position of the observer: move the position of the right-hand controller while pressing the right grip button or move the observer’s head in real space (VR goggle detects movements).

(iv) Reset the rotation of the specimen: press the X or reset buttons in the panel.

(v) Change the interested view of the specimen: press the left trigger button. The aim is to change the *Z*-, *X*- and *Y*-axis projections, indicated by *XYZ*, *YZW*, *ZWX* and *WXY* in VR space, sequentially.

(vi) Details of the parameters can be determined from the menu button on the left panel.

Frame: select the frame corresponding to the energy of the incident X-rays.

Minimum: the minimum value of intensity for the contrast.

Maximum: the maximum value of intensity for the contrast.

Size: adjust the size of the specimen in VR space.

Intensity: multiply the value by the intensity of the image.

Show all frames: shows the data in 4D, as explained in Section 3.3.

Show all projections: select the non-interesting projections drawn in VR space.

Blend: select alpha blending (or not).

Clip distance: the distance for drawing when the observer comes near the specimen.

Highlight: highlights the current frame.

Steps: number of samples for volume ray-casting.

Smoothing radius: the size of binning voxels for extraction of spectra.

Viewing the 3D volume data through the *4D-XASView* system offers several advantages for understanding the 4D XAFS data. Conventionally, 3D volume data are displayed on a monitor (2D), making it difficult to understand their geometric and topological features. For example, certain microstructures within a region may be obscured by others, necessitating the rotation of the specimen to bring them to the forefront. Mental reconstruction is often required to combine these views and understand the microstructure observed. However, with *4D-XASView*, we can directly view the microstructure observed in 3D within VR space, gaining clear insights into its detailed microstructures, even in cases involving complex or hierarchical structures.

Furthermore, observers can immerse themselves inside the specimen within VR space, exploring and examining microstructures to identify unique features [refer to Figs. 3 ▸(*b*) and 3 ▸(*c*), and see Movie S1 of the supporting information]. During our observations, we discovered an ‘island-shaped’ microstructure exhibiting a different chemical state (magnetite: Fe_3_O_4_), which was confirmed using the 3D-spec. mode discussed in Section 3.2. This mode proves highly effective for understanding microstructures in various systems, including pores or hollows commonly found in porous materials or catalyst substrates.

### 3.2. 3D-spectroscopy mode: extraction of XAFS spectra at a specific location

In this mode, we can observe the XANES or extended-XAFS spectrum corresponding to a selected region from the 3D volume data within VR space. The XAFS spectrum is displayed on selection of the position of interest, which is achieved using the trigger on the right-hand controller. To choose a position, the right hand is maneuvered inside the specimen in the VR environment. The binning size is adjusted using the ‘Smoothing radius’ setting on the panel.

**Figure 5:**
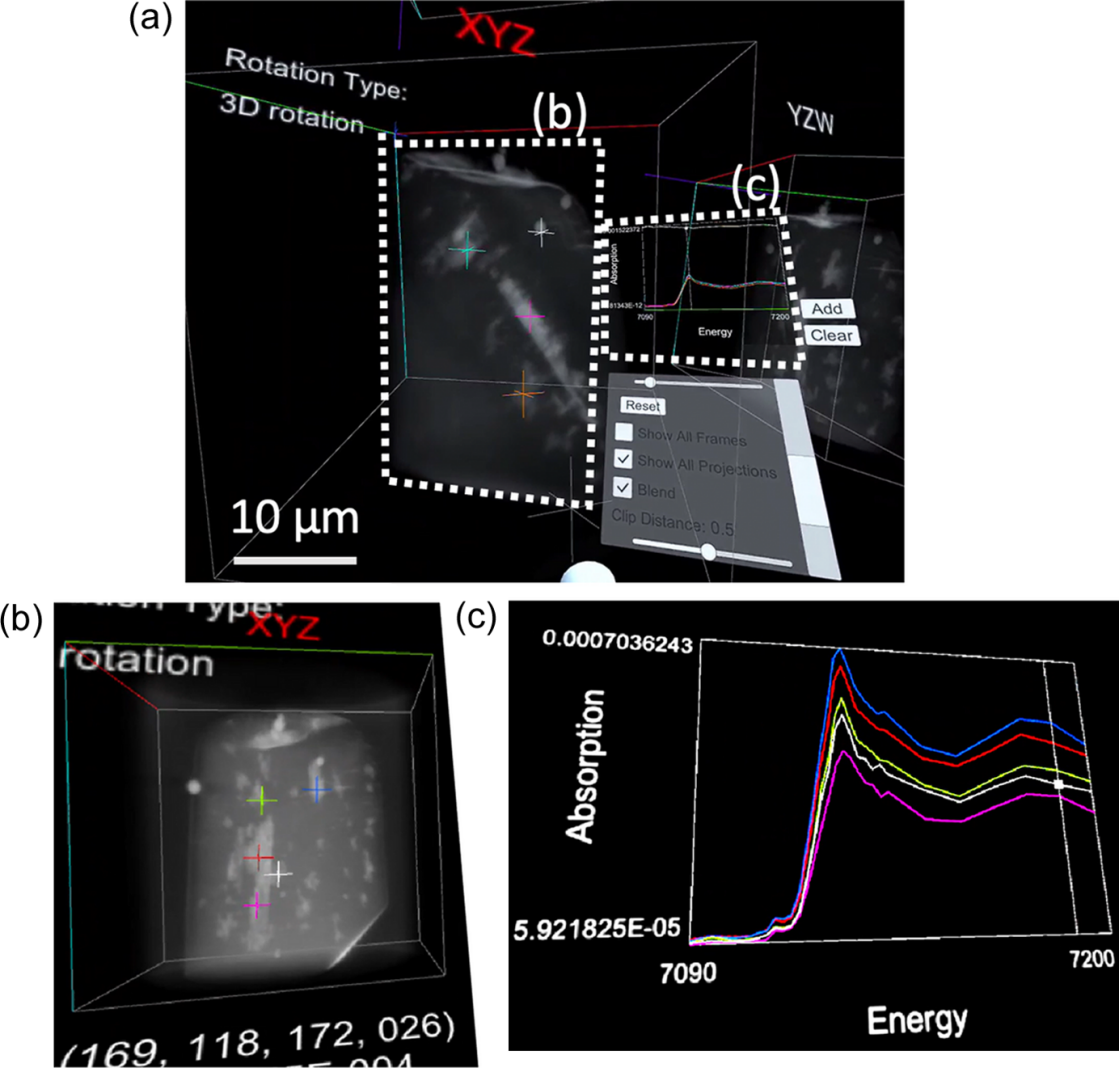
Views of the 3D-spec. mode in VR space using *4D-XAFSView*. (*a*) Overview of the VR views. (*b*) Specification of positions of interest in 3D within the specimen. (*c*) XANES spectra corresponding to the specified positions [colored cross marks in (*b*)].

Moreover, XAFS spectra from various positions of interest can be compared, as depicted in Fig. 5 ▸.

(i) Select the position of interest in the specimen: place the right-hand controller inside the specimen and press the right trigger button. The spectrum of the selected position is shown in white in the figure panel and the white cross in the specimen.

(ii) Record the selected spectrum for comparison: the selected position or spectrum is recorded by the A button on the right-hand controller or the ‘Add’ panel on the right-hand side of the figure panel. The color of the cross for the identification of the position in the specimen and the spectrum in the figure changed sequentially.

(iii) Clear the spectra: press the ‘Clear’ panel on the right side of the figure panel.

Fig. 5 ▸ illustrates typical views of the 3D microstructure of the specimen at an X-ray energy of 7190 eV, approximately 79 eV above the Fe *K*-edge, in conjunction with the XAFS spectra in the selected region (indicated by crosses) within VR space. The bright microstructures in Fig. 5 ▸ indicate areas of high absorbance, reflecting high electron density (typically containing high-*Z* elements).

By utilizing the 3D-spec. mode, we can compare the chemical states from XAFS spectra and identify specific elements because each voxel contains spectroscopic data in the 4D XAFS data. In Fig. 5 ▸, regions highlighted in pink, light blue, red, light green and white contain iron with differing chemical states or Fe(II)/Fe(III) ratios as discussed in Section 4.

In materials with complex microstructures, the chemical states of regions exhibiting specific geometric features are often different from those of other regions owing to various reactions, including redox processes (Kimura *et al.*, 2018 ▸) and diffusion. Traditionally, 4D XAFS data are processed by selecting slice data from the corresponding 3D volume data and analyzing the relationship between 2D microstructures and their co-existing phases that are determined by analysis of XAFS spectra for corresponding pixels. Typically, the XAFS spectra of each voxel are analyzed using reference materials through linear combination fitting. However, simultaneous 3D viewing and confirmation of XAFS spectra are essential for identifying geometric features such as dendritic, pillar-type, and plate-like structures and their chemical states. Thus, the 3D-spec. mode represents one of the most powerful and beneficial aspects of the *4D-XASView* system.

Unlike conventional analyses, our proposed method enables the extraction of regions containing characteristic 3D microstructures within VR space, allowing direct confirmation of their chemical states in the XAFS spectrum (Fig. 5 ▸).

### 3.3. 4D mode: visualization of the 4D representation

The 3D-spec. mode discussed earlier serves as a potent tool, even though it aligns with traditional human research methods: segmenting voxels based on reference materials and subsequently assessing the geometric and topological features of each segmented region. This conventional approach treats the (*x*, *y*, *z*) space and the energy space separately, effectively reducing the energy space through segmentation under the assumption that the specimen shares chemical states similar to the reference materials. Consequently, this approach may overlook intrinsic information embedded in 4D or higher multidimensional data. For instance, in cases wherein trigger sites exhibit chemical states significantly different from those of the reference materials, we require an approach that is capable of directly extracting information from 4D or higher multidimensional space without relying on assumptions about the chemical states using reference materials.

To address this need, the 4D mode offers direct visualization of the 4D data representation within VR space. In the 4D mode, the images in the VR environment can be manipulated using two motion controllers, offering a broader range of options, as outlined below (also refer to Movie S2 of the supporting information):

(i) Change the rotation mode with two presentations: (*a*) 3D rotation or (*b*) 3D rotation + 4D rotation by translation – press the left stick.

(ii) 4D Rotation of the specimen: the translation of the left-hand controller by pressing the left grip button when in ‘3D rotation + 4D rotation by translation’ rotation mode. In the case of the 4D representation, the view of the specimen is different from that obtained using the 3D view mode.

(iii) Show the 3D volume view of the specimen at the same time: press the right stick button.

**Figure 6:**
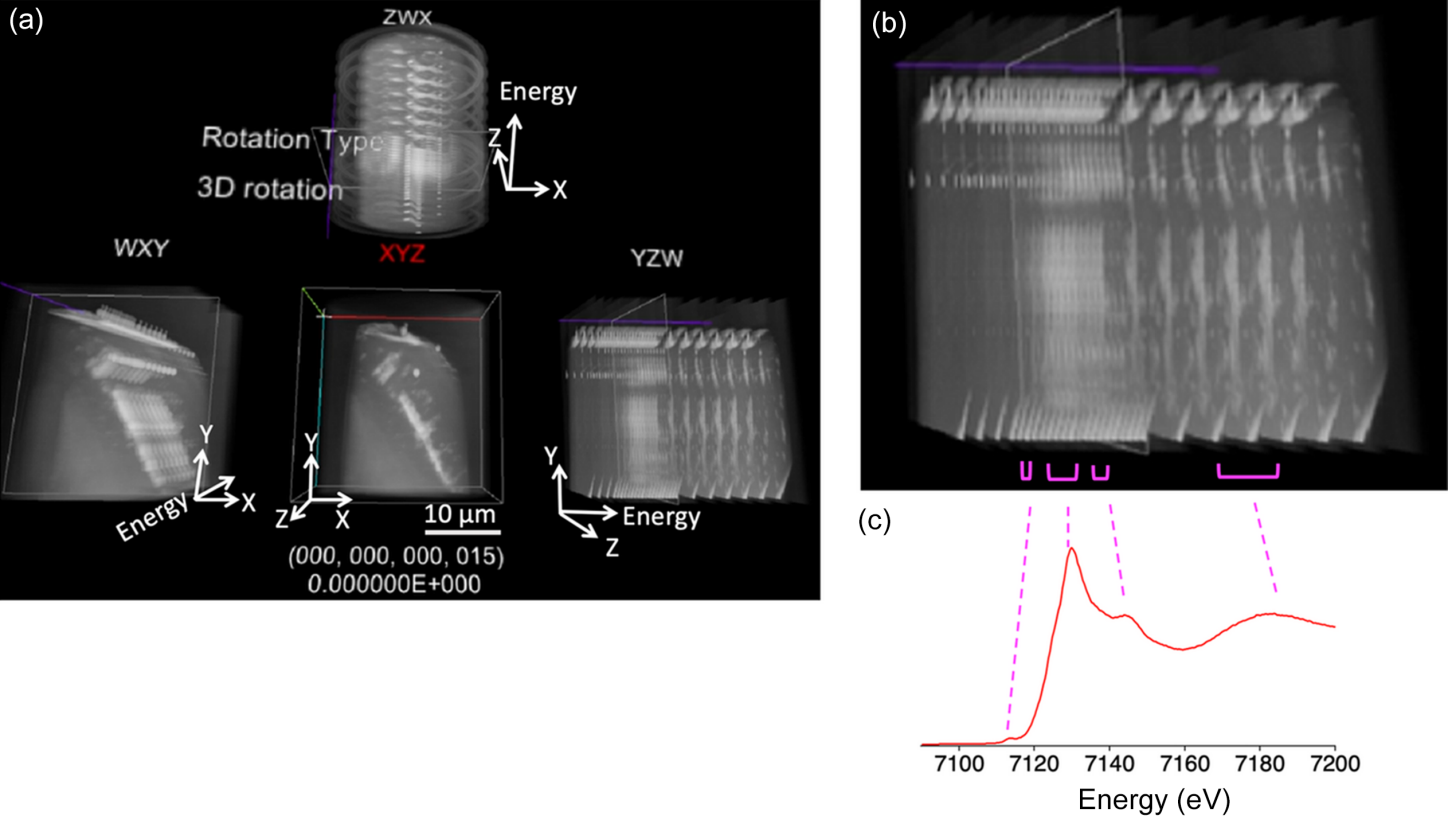
(*a*) Views of the 4D mode in VR space using *4D-XASView* with the 3D rotation presentation. (*b*) Magnified image of the right image in (*a*). (*c*) XANES spectrum of Fe_3_O_4_.

Fig. 6 ▸ presents a typical example of viewing the specimen within the energy range 7090–7200 eV with the ‘show all frames’ option selected. Stacks of projections in three directions are depicted in VR space. In the *YZW* projection shown in Fig. 6 ▸, the values along the *W* axis (corresponding to the energy of the incident X-ray) increase towards the right. The line component within the specimen appears brighter or white in the image stack, indicating the presence of iron species. Conversely, areas devoid of iron species remain mostly unchanged.

In Fig. 6 ▸(*a*), all the projections are displayed in VR space. However, each projection merely represents a 3D depiction achieved by collapsing one of the spatial or energy axes and thus remains insufficient as a direct 4D representation. Though the ‘3D rotation’ mode offers conventional 3D rotational views, another rotation mode, namely ‘3D rotation + 4D rotation by translation’, provides a 4D representation through 4D rotations. These 4D rotations provide smoothly transitioning projections that offer a diagonal perspective in 4D, allowing simultaneous observation of each spatial and energy axis. This approach facilitates interactive exploration of the 4D structure constituted by the 4D XAFS data (see Movie S2 of the supporting information). Unlike the 3D presentation, this mode can be used to view and understand 4D XAFS data directly, although some time is required to become familiar with this presentation.

## 4. Discussion

We successfully visualized and comprehended XAFS data in 4D (3D space + energy) using VR space through the use of *4D-XASView*, developed based on the *Polyvision* program. Herein, we explored the advantages and potential future aspects of the system, focusing on the results obtained by viewing the 4D XAFS data of the specimen using *4D-XASView* (refer to Figs. 3 ▸, 5 ▸ and 6 ▸).

**Figure 7:**
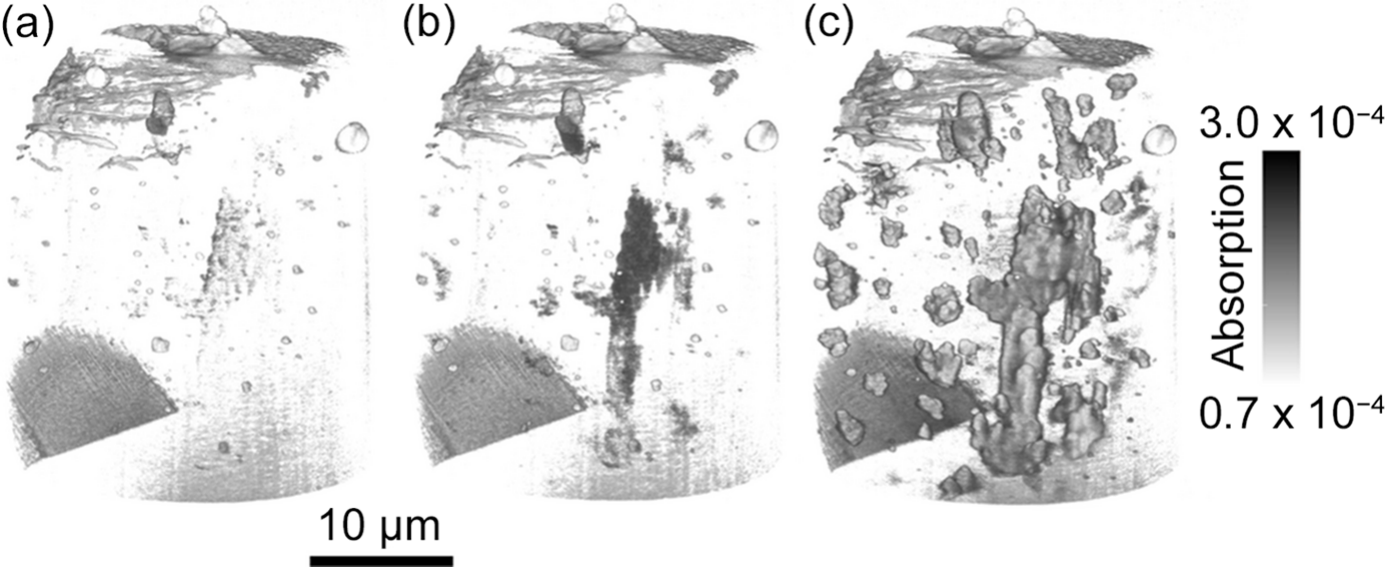
3D images of X-ray absorption at *E* values of (*a*) 7090 eV, (*b*) 7112 eV and (*c*) 7128 eV of the sample.

**Figure 8:**
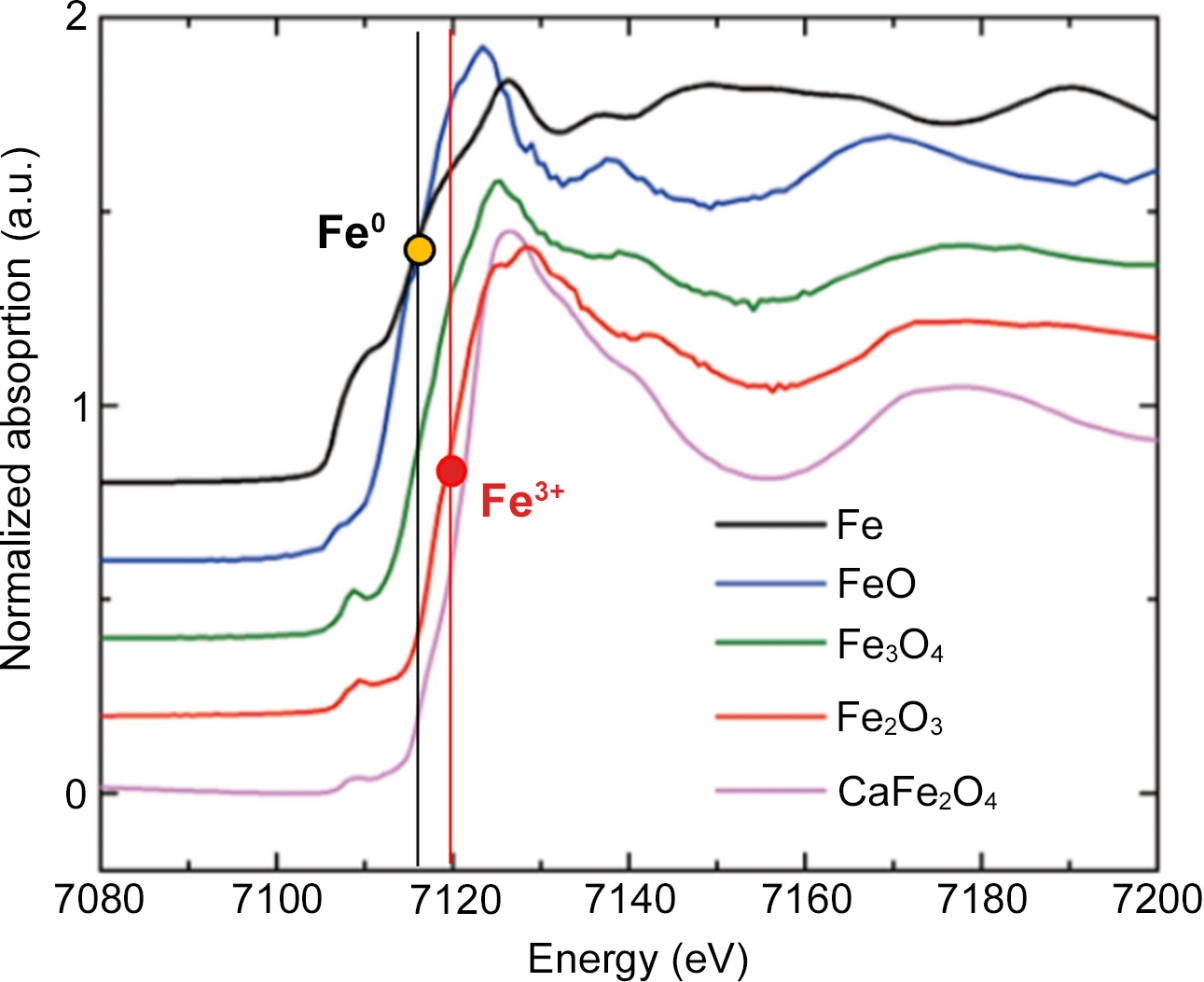
Typical *K*-edge XANES spectra of iron oxides and metal.

When attempting to view and understand 4D data using a conventional 3D viewer, we must condense the dimensions from 4D to 3D. One of the simplest methods involves the comparison of 3D images with varying X-ray energies. Fig. 7 ▸ depicts 3D images of X-ray absorption at *E* = 7090, 7112 and 7128 eV of the sample. Areas exhibiting high-absorption levels, which exceed that of the Fe *K*-edge, at 7112 and 7128 eV indicate iron accumulation. To identify the chemical states of iron in these regions, we need to extract XAFS spectra and compare them with those of reference materials (Fig. 8 ▸). This process, typically performed using specialized software, is often performed as a batch process and lacks interactivity. Specification of the region of interest in 3D is especially challenging when dealing with specimens featuring complex microstructures. Moreover, reducing the dimensionality from 4D to 3D based solely on XAFS analysis may result in information loss, hindering the utilization of various modern techniques for multidimensional data analysis.

However, we can explore and analyze 4D data interactively and intuitively using the *4D-XASView* system developed, as depicted in Figs. 3 ▸, 5 ▸ and 6 ▸, especially owing to the ability of the system to facilitate direct visualization of 3D specimens and simultaneous extraction of XAFS spectra. A 2D display can display only part of the specimen’s 3D image, such as cross-sectioned images or perspective images from specific directions, and thus, the observer must construct a 3D image in their brain for full comprehension. Our method presents the 3D specimen directly to the observer, enabling intuitive operations. Users can select points of interest for comparing XAFS spectra through intuitive actions such as navigating inside the specimen or rotating the 3D representation [Figs. 3 ▸(*b*) and 3 ▸(*c*)].

In the analyzed specimen, magnetite appears aligned in plates but exhibits irregular shapes as aggregates of fine grains. The *4D-XASView* system elucidated this unique microstructure where magnetite nucleated and grew within fractures induced by volume-increasing reactions (Shimizu & Okamoto, 2016 ▸; Yoshida *et al.*, 2020 ▸), which is important for understanding the continuous transportation of seawater and for the oxidation of Fe(II) in olivine to Fe(III) in magnetite or serpentine to generate H_2_ during serpentinization. The *4D-XASView* program enabled us to identify the formation of magnetite and analyze its 3D morphology, offering essential insights into the mechanism of seawater infiltration in oceanic lithosphere rock.

Another significant advantage of the *4D-XASView* system is its ability to select specific positions even within complex microstructures for viewing spectra (Fig. 5 ▸). For example, cracks formed during the reduction of Ca–Fe–O oxides originate from positions where the microstructures exhibit specific shapes (Kimura *et al.*, 2018 ▸), or cracks initiate from particular locations in carbon-fiber-reinforced plastics (Kimura *et al.*, 2019 ▸, 2022 ▸). Therefore, simultaneous viewing of specific microstructures and chemical states using the *4D-XASView* system proves highly effective in investigating trigger sites in materials.

Another crucial advantage of the *4D-XASView* system, which is quite distinctive, lies in its ability to offer fundamental insights into the analysis of 4D data based on shape or topological approaches (Kimura *et al.*, 2018 ▸; Obayashi *et al.*, 2018 ▸). Various informatic approaches such as clustering, neural networks and others (Bishop, 2006 ▸) have been extensively utilized for analyzing multidimensional data. However, most of these methods are black-box approaches, where the optimization process is neither open nor clear; therefore, it can be challenging to interpret their findings within scientific or materials science contexts. In contrast, with 4D XAFS data, each axis has a physical meaning: space (*x*, *y*, *z*) and X-ray energy, while the shape or topological features within the heterogeneity in 4D should also carry physical or materials science implications. Therefore, analyzing 4D data in terms of shape or employing a topological approach can be considered a white-box approach. In this scenario, the optimization process is clear and can be understood within the scientific or material science knowledge. This approach allows us to validate the identified trigger sites effectively (Kimura *et al.*, 2018 ▸; Obayashi *et al.*, 2018 ▸).

The 4D mode of *4D-XASView* precisely represents unique features inherent in these types of 4D data. Distinct geometric and topological attributes within the space that includes the energy axis can be observed and investigated (Fig. 6 ▸). Subsequently, we can analyze the heterogeneity in 4D using mathematical or topological techniques like persistent homology. For example, in the analyzed specimen, brighter regions can be observed in the space that includes the energy axis. Fig. 6 ▸(*b*) shows the image in the *Y*–*Z*–energy space, and brighter regions indicated by pink brackets are visible. In other words, high absorption values are detected in some island-shaped regions in the (*X*–)*Y*–*Z*–energy space. Thus, the analysis of 4D XAFS data in 4D space can reveal the trigger sites in the (*x*, *y*, *z*) space, corresponding to a shape or microstructure, and in the energy space, corresponding to an XAFS spectrum. The spectrum of the sample analyzed in this study corresponds to the reference material: Fe_3_O_4_ [Fig. 6 ▸(*c*)]. However, in general, the shape features of the XAFS spectra of trigger sites, which are different from those of reference materials, may be suggested through the analysis of 4D XAFS data in 4D space.

This process aids in pinpointing the location and energy state related to trigger sites without relying on assumptions about reference materials or reported chemical states. This approach is crucial for extracting comprehensive information from large and multidimensional datasets, transcending previous material knowledge or understanding. The energy dimension in 4D XAFS data (3D space + energy) differs from the 3D space. This distinction applies to other multidimensional data obtained from techniques like 3D XRD, wherein 4D data consist of 3D space + diffraction angle (or scattering vector). Thus, *4D-XASView* serves as a versatile platform for analyzing 4D XAFS and other multidimensional data using various mathematical or informatic approaches.

One limitation of the *4D-XASView* system is its high demand for computational power to handle massive datasets, particularly for smooth functioning. In this study, data reduction was performed through binning as a preprocessing step. Therefore, an upgrade of the *4D-XASView* system is essential, incorporating informatics techniques for handling multidimensional data, which may include hardware upgrades such as a graphics processing unit.

A future aspect of the analytical approach using the *4D-XASView* system involves its adaptation to handle 5D data [3D space + energy + time (or reaction)]. This development is feasible due to advancements in spectromicroscopy with XAFS-CT or TXM. Analyzing 5D data enables us to identify unique geometric and topological features within the 5D space, aiding the prediction of trigger sites that govern macroscopic properties based on materials and chemical states. Our proposed approach using *4D-XASView* is expected to serve as a vital platform for addressing this challenge via intuitive 5D data visualization and operations.

## 5. Conclusions

We developed a novel approach and a computational program – *4D-XASView* – by building on the *Polyvision* program, to visualize XAFS data in 4D using VR technology. With *4D-XASView*, we can effortlessly view and analyze data using VR goggles, gaining comprehensive insights into microstructural details through interactive rotations and zooming features. Furthermore, the program facilitates the extraction of XAFS spectra data by pinpointing specific positions or regions in 3D, thus enabling efficient identification of specialized locations that dictate macroscopic properties in materials. Our innovative method establishes a new platform for analyzing 4D or 5D XAFS data. This approach is planned to be extended to various multidimensional datasets, including 3D microstructures as well as other 3D datasets, such as spectroscopic, diffraction and various-property (magnetic, mechanical *etc*.) datasets.

## Supplementary Material

Recording of the image using VR goggles during the analysis of 4D XAFS data in (a) 3D volume data mode, (b) 3D spectroscopy mode and (c) 4D mode in `3D rotation' presentation. Recording using VR goggles; the controller is also included as an insert. DOI: 10.1107/S1600577524011226/vy5033sup1.mp4

Recording of the image using VR goggles during the analysis of 4D XAFS data in (a) 3D volume data mode, (b) 3D spectroscopy mode and (c) 4D mode in `3D rotation' presentation. Recording using VR goggles; the controller is also included as an insert. DOI: 10.1107/S1600577524011226/vy5033sup2.mp4
